# Differential Effects of Lateral and Medial Entorhinal Cortex Lesions on Trace, Delay and Contextual Fear Memories

**DOI:** 10.3390/brainsci12010034

**Published:** 2021-12-28

**Authors:** Brett S. East, Lauren R. Brady, Jennifer J. Quinn

**Affiliations:** Department of Psychology and Center for Neuroscience & Behavior, Miami University, 90 N. Patterson Ave., Oxford, OH 45056, USA; bsej87@gmail.com (B.S.E.J.); bradylr@miamioh.edu (L.R.B.)

**Keywords:** expression, consolidation, learning, memory, rat

## Abstract

The entorhinal cortex (EC), with connections to the hippocampus, amygdala, and neocortex, is a critical, yet still underexplored, contributor to fear memory. Previous research suggests possible heterogeneity of function among its lateral (LEC) and medial (MEC) subregions. However, it is not well established what unique roles these subregions serve as the literature has shown mixed results depending on target of manipulation and type of conditioning used. Few studies have manipulated both the LEC and MEC within the same experiment. The present experiment systematically manipulated LEC and MEC function to examine their potential roles in fear memory expression. Long-Evans rats were trained using either trace or delay fear conditioning. The following day, rats received an N-methyl-D-aspartate (NMDA)-induced lesion to the LEC or MEC or received a sham surgery. Following recovery, rats were given an 8-min context test in the original context. The next day, rats were tested for tone freezing in a novel context with three discrete tone presentations. Further, rats were tested for hyperactivity in an open field under both dark and bright light gradient conditions. Results: Following either LEC or MEC lesion, freezing to context was significantly reduced in both trace and delay conditioned rats. LEC-lesioned rats consistently showed significantly less freezing following tone-offset (trace interval, or equivalent, and intertrial interval) in both trace and delay fear conditioned rats. Conclusions: These data suggest that the LEC may play a role in the expression of a conjunctive representation between the tone and context that mediates the maintenance of post-tone freezing.

## 1. Introduction

A detailed literature exists demonstrating a role for the hippocampus in the acquisition and expression of some forms of memory, but considerably less attention has been devoted to one of its major contributing structures: the entorhinal cortex (EC). Given the reciprocal connections of the EC with both the hippocampus and the medial prefrontal cortex [1,2,3,4], it was historically considered a relay station for information to and from the cortices and the hippocampus. However, recent studies suggest independent roles for discrete subregions of the EC [5,6], but the data have been mixed.

There are a number of potential explanations for the apparent discrepancies. Some studies have targeted the entire EC with no distinction being made between the lateral or medial EC subregions [7,8,9,10]. Others have manipulated a single subregion without assessing potential contributions of the other subregion [11]. In some instances, the EC lesion extent is quite large and encompasses significant portions of the ventral hippocampus [7,9], while other lesions have been restricted to a specific subregion of the EC [12]. Another important consideration is the type of conditioning being used, with some using trace and/or delay fear conditioning [10], some using trace and/or delay eyeblink conditioning [11], and some using unsignaled contextual fear conditioning [7,8,9,12].

For trace and delay fear conditioning, pre-training neurotoxic lesions of the entire entorhinal cortex produced impairment in trace, but not delay, conditioning [10]. Similarly, targeted inhibition of calcium calmodulin-dependent protein kinase II (CaMKII) signaling in entorhinal cortex layer III input disrupts trace, but not delay, fear conditioning [13]. Further, temporally-specific optogenetic inactivation of medial entorhinal cortex (MEC) layer III input showed similar effects on trace, but not delay, fear conditioning. Inactivation during the trace interval and shock produced a deficit, but there was no deficit when inactivation was restricted to only tone-on periods. This suggests that MEC may be necessary for maintaining the memory for tone during the post-tone period, but not during the tone itself [14,15].

A number of studies using eyeblink conditioning have provided additional support for subregion specificity in entorhinal cortex function. Lesions and pharmacological manipulations suggest that lateral entorhinal cortex (LEC) is important for the acquisition of trace eyeblink conditioning [16,17]. Interestingly, inactivation of LEC, but not MEC, prior to testing yields an impairment in trace, but not delay, eyeblink memories [18,19]. It is important to note that contextual fear conditioning may also depend upon the LEC, although the literature is currently conflicting [7,8,9,12].

It has been proposed that the medial entorhinal cortex may be more involved in spatial memory and spatial navigation (though contextual fear, specifically, remains less clear cut), whereas lateral entorhinal cortex may be more involved in temporal association, specifically the ability to link two different stimuli (e.g., tone and footshock) even when they are separated in time, as in trace conditioning [6]. In the present experiment, we systematically manipulated the EC subregion being targeted (lateral versus medial) and the type of fear memory expression being assessed (trace tone versus delay tone versus context). Another key difference between the present experiment and previous reports is the analysis of fear expression (freezing) throughout the entire tone test. Previous studies have restricted their analyses to responding during the tone and/or trace interval. In the current study, we analyzed freezing during each tone, each trace interval (or equivalent time period for delay conditioned rats), and throughout each intertrial interval. This is important, as we have learned from the hippocampus literature, where lesions were initially reported to have an effect on trace or context, but not delay, conditioning (e.g., see [20] for review). However, subsequent reports revealed that lesions of the hippocampus produce deficits in delay fear memory expression when freezing is assessed following tone offset [20,21,22]. Thus, different aspects of the fear memory seem to be revealed by different periods of measurement.

Finally, we assessed activity levels under both dark and bright light gradient conditions in an open field. Previous studies have shown that hyperactivity can result from lesions of the hippocampus (e.g., [23,24]). Since the behavioral measure used in the present experiment is freezing, it is important to ensure that lesions of the LEC or MEC subregions do not produce hyperactivity, which could interfere with the performance of freezing. The open field procedure used in the present experiment has proven to be sensitive to changes in basal activity levels in rats [25].

## 2. Materials and Methods

### 2.1. Animals

Sixty-one naïve, male, Long-Evans rats (~90 d of age; bred in house) were used. Rats were pair-housed on a 14:10 h light-dark cycle, given ad libitum access to food and water, and handled for five consecutive days before surgery. All experimental procedures were performed during the light cycle and approved by the Miami University Institutional Animal Care and Use Committee in accordance with the NIH Guidelines for the Care and Use of Experimental Animals.

### 2.2. Intracranial Surgery

**Figure 1:**
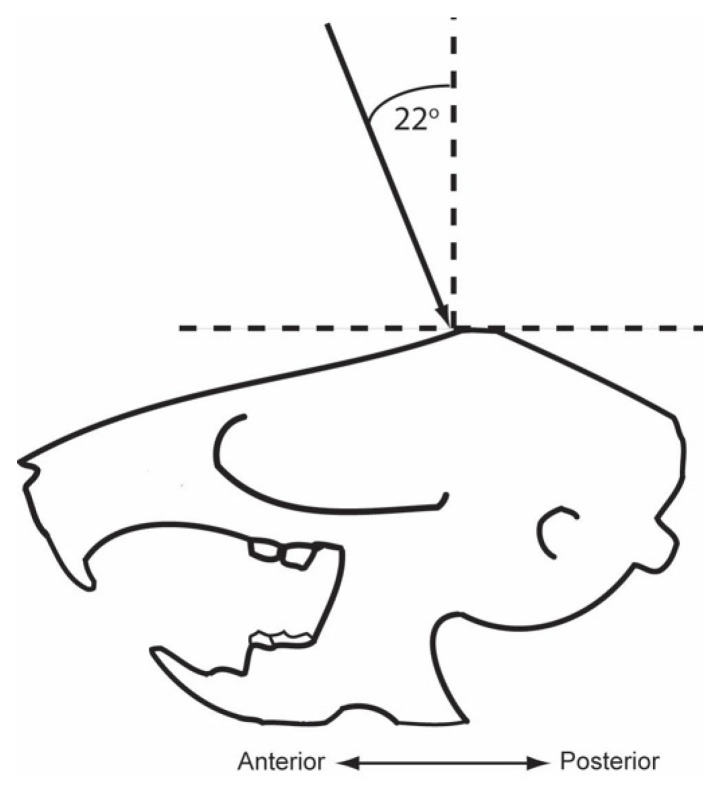
A schematic demonstration of the angle of entry used for MEC lesions.

Following training, rats received a lesion of lateral EC (LEC), medial EC (MEC), or a sham surgery. Rats were anesthetized via 5% isoflurane (Vedco) inside an induction chamber before being placed into the stereotaxic apparatus where they were maintained at 2–3% isoflurane. Incisions down the midline of the skull were made and expanded to expose lambda and bregma which were subsequently used to level the skull surface. Surgery coordinates were measured with respect to bregma and drilled. Stainless steel tubing connected to a 10 µL Hamilton syringe via polyethylene tubing was used for drug delivery. Lesions consisted of manually infusing 0.1 µL/site NMDA (20 µg/µL) [26]. After infusion, injectors remained in place for an additional 2 min to allow for diffusion. Rats in the sham surgery condition received identical surgical treatment except that no injector was lowered and no drug infused. The following coordinates (relative to bregma) in Table 1 were used to lesion the LEC at 4 bilateral sites or the MEC at 3 bilateral sites (see Table 1). Sites for the MEC were approached with the needle oriented 22° posteriorly (see Figure 1).

Following surgery, rats received subcutaneous injections of 3 mL 0.9% saline, 5 mg/kg Rimadyl (5 mg/mL), and 0.2 mL diazepam before being placed in a heated recovery cage and allowed to become ambulatory before being returned to a clean homecage. Additionally, post-operative care consisting of 3 mL 0.9% saline and 5 mg/kg Rimadyl (5 mg/mL) was administered subcutaneously for two days following surgery, along with weight monitoring and handling for five consecutive days.

### 2.3. Apparatus

Four conditioning chambers (32.4 cm W × 25.4 cm D × 21.6 cm H; Med Associates, Inc., Fairfax, VT, USA) were used for fear conditioning and context testing. Both the front door and ceiling were made of clear Plexiglas, a white plastic back wall, and sides made of aluminum. Floors consisted of 19 equally spaced stainless steel rods which were connected to a shock generator and scrambler (Med Associates, Inc., Fairfax, VT, USA). A stainless steel pan was located below the grid floor and was coated with a 1:1 mixture of water and artificial vanilla flavoring to provide context odor. The chamber was illuminated by a 125 lux white light with fans providing a background noise of 68 db. Between rats, chambers were cleaned with a 5% NaOH solution and a fresh vanilla mixture placed into the pan.

A novel context for tone testing consisted of 4 separate chambers of equal size and shape. However, these chambers contained a solid white Plexiglas floor and a black triangular insert placed over the floor. Additionally, the pan below the floor contained white vinegar. This provided novel visual, tactile, and olfactory elements to distinguish this context from the training context. Finally, testing in these chambers was performed in complete darkness using cameras sensitive to near-infrared lighting (0 lux).

For all fear conditioning and testing, rats were monitored by a progressive scan video camera with visible light filter (VID-CAM-MONO-2A; Med Associates, Inc., Fairfax, VT, USA) connected to a computer running VideoFreeze software (Med Associates, Inc., Fairfax, VT, USA) designed for the automated assessment of motion, including defensive freezing [27].

Open field was conducted in a black, plastic container with dimensions of 101 cm × 54.6 cm × 45.4 cm. At the far end of one side of the chamber were two lamps each containing a 13 watt soft white CFL bulb. The lamps were attached above the chamber facing downward to create a light gradient that dissipated at the midpoint of the chamber. The light measured 1700 lux at the brightest portion of the chamber, 25 lux at the center, and 5 lux at the end furthest from the lamp. An overhead camera recorded the session and data were analyzed using ANY-maze tracking software (Stoelting Co., Wood Dale, IL, USA). For the purpose of scoring crossovers, the floor of the chamber was divided into 8 equally sized squares. Crossovers were automatically calculated by the ANY-maze software whenever 75% of a rat’s body entered a zone.

### 2.4. Fear Conditioning

Rats were trained using one of two types of fear conditioning: trace or delay. Trace fear conditioning consisted of 10 trials following a 180 s acclimation period. Each trial had a 16-s tone (2 kHz, 75 dB), a 28-s “stimulus-free” trace interval followed by a 2-s footshock (0.9 mA). Trials were separated by a 256 s intertrial interval (ITI) from tone onset to tone onset. For delay fear conditioning, rats had a period of 180 s for acclimation before beginning a series of 3 trials in which a 16-s tone (2 kHz, 75 dB) coterminated with a 2-s, 0.9 mA footshock. The ITI was 256 s. The difference in training trials for trace vs. delay (10 vs. 3 trials, respectively) was based on previous data from our lab. Trace conditioning is slower to acquire and 10 trials produce significant freezing to tone and context. However, delay conditioning is considerably faster, and in order to produce comparable levels of tone and context freezing, three trials were used.

### 2.5. Fear Testing

After 7 days of surgical recovery, rats received two test sessions. The context test consisted of placing the rat back into the original training context and freezing was measured over an 8-min period. No tones or footshocks were presented. The tone test occurred in a novel context and consisted of a 180-s baseline period followed by three discrete presentations of the tone used during conditioning, separated by 256 s ITI.

### 2.6. Open Field

Rats were tested in the open field the next day following tone test. Rats were kept in home cages in an adjacent room and brought to the open field in transport cages. At the start of each trial, rats were placed in the center of the open field with both lamps turned off. After four minutes, the two lamps were turned on to create the light gradient. After four additional minutes, the rats were removed and brought back to their home cage. Between rats, the transport cages were cleaned with 5% NaOH and the open field was cleaned with Simple Green (Sunshine Makers, Inc., Huntington Beach, CA, USA).

### 2.7. Histology

Upon completion of behavioral testing, rats were administered 0.3 mL Euthasol (Virbac Animal Health, Inc., Westlake, TX, USA; 390 mg/mL pentobarbital + 50 mg/mL phenytoin) before being transcardially perfused with PBS-A followed by 4% paraformaldehyde. Brains were extracted and stored in 20% glycerol prior to slicing. Coronal sections (40 µm) were collected and stained using thionin (Sigma-Aldrich, St. Louis, MO, USA).

In a small subset of lesioned brains, we confirmed our impressions from the thionin-stained tissue using immunohistochemistry targeting both GFAP (Glial Fibrillary Acidic Protein) and NeuN (Neuronal Nuclei marker) [28,29]. Slices were given a series of washes in 0.1 M PBS followed by overnight incubation in 0.1 M PBS, 0.2% Triton-X solution, blocked with normal donkey serum, and given an incubation for 48 h in primary antibody: Mouse anti-NeuN (Millipore MAB377) and chicken anti-GFAP (Abcam64674) diluted in 0.1 M PBS. Following another set of washes, sections were then incubated for 2 h in AlexaFluor conjugated antibodies directed towards the primary host antibody (Alexa Fluor 555 Donkey Anti-mouse, Life Technologies A-31570, Alexa Fluor 488 Donkey Anti-chicken, Jackson Immuno 703-545-155). Sections were then rinsed, mounted, and coverslipped with Vectashield hardest antifade mounting medium with DAPI (Vector Laboratories, H-1500). Images were captured using an Olympus AX-70 Research System microscope.

## 3. Results

### 3.1. Histological Verification

**Figure 2:**
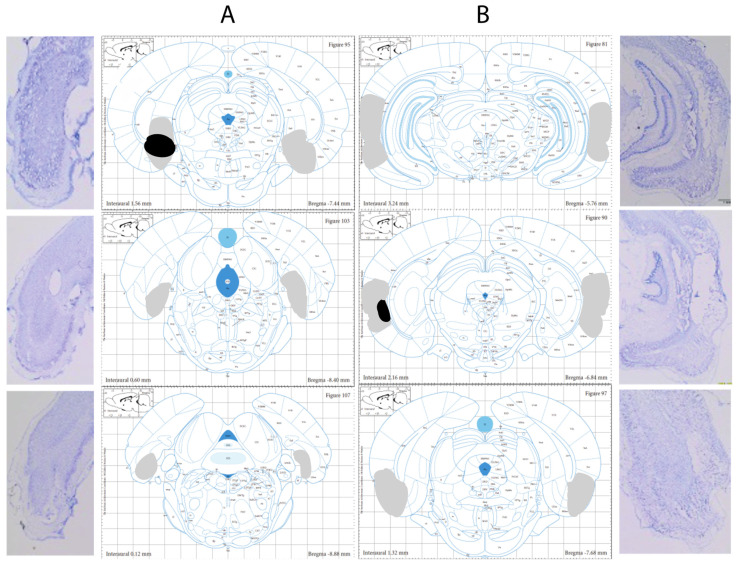
Atlas plates demonstrating the minimal (black) and maximal (grey) lesion extents for MEC (**A**) and LEC (**B**) lesions, along with representative photomicrographs (Reprinted with permission from Ref. [30]. Copyright 1998 Academic Press: Cambridge).

Figure 2 shows atlas plates indicating the lesion extent for the animal with the least amount of damage (black) and the animal with the greatest amount of damage (grey) for MEC (Figure 2A) and LEC (Figure 2B) rats. Eight MEC-lesioned and five LEC-lesioned animals were excluded from all analyses due to insufficient bilateral damage (*n* = 4), extensive damage (*n* = 5), or placement that was too dorsal (*n* = 4). For all statistical analyses, the number of animals in each condition is: Trace-Sham, 10; Trace-LEC, eight; Trace-MEC, eight; Delay-Sham, eight; Delay-LEC, six; Delay-MEC, eight. For those animals included in subsequent analyses, the percentage of total area damaged by the lesion was calculated at each anterior-posterior level for each animal (three per rat; left and right). In general, the extent of damage was greatest more anteriorly for both MEC and LEC lesions. The average percent of MEC damaged from anterior to posterior was: 47% (AP: −7.44 mm), 15% (AP: −8.40 mm), and 14% (AP: −8.88 mm). The average percent of LEC damaged from anterior to posterior was: 60% (AP: −5.76 mm), 55% (AP: −6.84 mm), and 35% (AP: −7.68 mm). The average MEC lesion extent (across all three levels) was 25% (range: 12–46%). The average LEC lesion extent (across all three levels) was 50% (range: 27–73%). These results from the thionin-stained tissue were confirmed by the GFAP/NeuN staining conducted in a small subset of lesioned brains.

### 3.2. Trace Fear Conditioned Animals—Context Test

**Figure 3:**
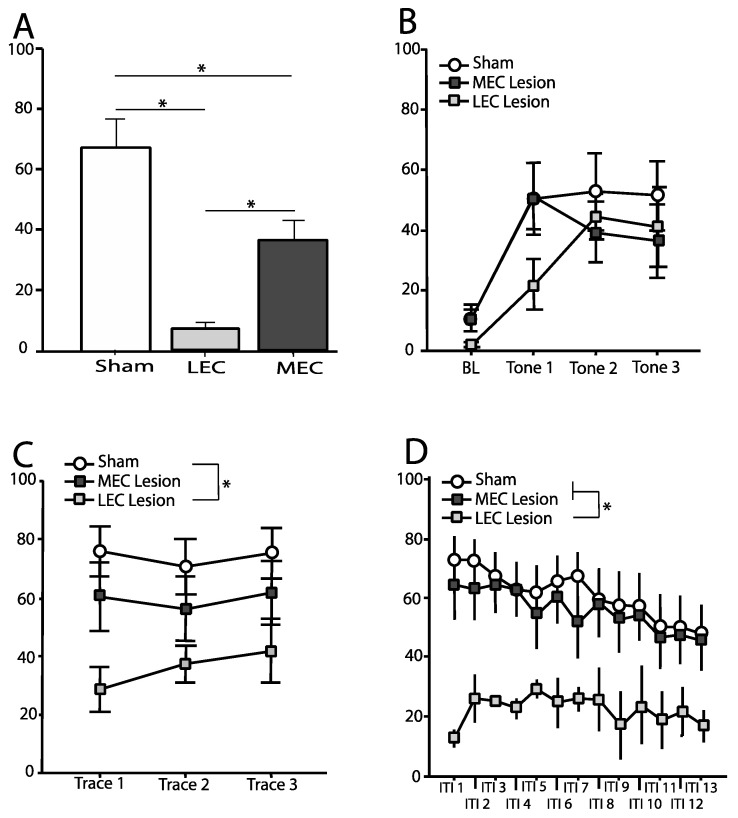
Mean (±SEM) percentage time spent freezing during the context test and tone test for rats trained using ***trace conditioning***. (**A**) Freezing during the entire 8-min context test. Both lesion groups show a deficit in freezing to context compared to controls. Additionally, LEC lesioned rats froze significantly less than MEC lesioned rats. (**B**) Baseline (BL) freezing taking place during the first 180 s of the tone test, prior to presentation of the first tone as well as freezing during each 16-s tone presentation. (**C**) Freezing during the 28-s trace interval following each tone presentation. LEC lesioned rats froze significantly less compared to control rats. (**D**) Freezing during each 212 s period following each trace interval divided into 16-s bins (with bin 13 being 20 s). LEC lesioned rats froze significantly less compared to both control rats and MEC lesioned rats. * indicates statistically significant group difference.

Freezing was measured during the entire 8-min context test. Figure 3A shows the mean (±SEM) percentage of time spent freezing during the context test for animals in each of the three conditions: Trace-Sham, Trace-LEC, Trace-MEC. A one-way ANOVA revealed a significant effect of condition on freezing to the context [*F*(2,23) = 18.53, *p* < 0.001]. Post-hoc comparisons using Fisher’s PLSD revealed that both Trace-LEC [*p* < 0.001] and Trace-MEC [*p* < 0.01] froze significantly less than Trace-Sham. Additionally, Trace-LEC rats froze significantly less compared to Trace-MEC rats [*p* < 0.05].

### 3.3. Trace Fear Conditioned Animals—Tone Test

#### 3.3.1. Baseline

Freezing was measured for the duration of the 180 s baseline period prior to the first tone and average percent freezing calculated. Figure 3B shows the mean (±SEM) percentage of time spent freezing during baseline. A one-way ANOVA revealed no significant effect of condition on freezing during the baseline period [*F*(2,26) = 2.54, *p* > 0.05].

#### 3.3.2. Tone

Freezing was measured for the duration of each 16 s tone. Figure 3B shows the mean (±SEM) percentage of time spent freezing during each tone. Repeated measures ANOVA revealed no significant differences across tones [*F*(2,44) = 0.17, *p* > 0.05] and no tone–condition interaction [*F*(4,44) = 1.12, *p* > 0.05]. In addition, there was no main effect of condition on freezing to the tone [*F*(2,22) = 0.95, *p* > 0.05].

#### 3.3.3. Trace Interval

Freezing was measured for the duration of each 28 s trace interval. Figure 3C shows the mean (±SEM) percentage of time spent freezing during each trace interval. Repeated measures ANOVA revealed no significant differences across trace interval [*F*(1,23) = 0.6, *p* > 0.05] and no trace interval–condition interaction [*F*(2,23) = 0.78, *p* > 0.05]. However, there was a significant main effect of condition on freezing during the trace interval [*F*(2,23) = 5.52, *p* = 0.01]. Post-hoc comparisons using Fisher’s PLSD revealed that Trace-LEC rats froze significantly less compared to Trace-Sham rats [*p* < 0.01], but Trace-MEC rats did not differ from controls [*p* > 0.05].

#### 3.3.4. Intertrial Interval

Freezing was measured for the duration of each 212 s period following each trace interval. The intertrial intervals were binned into 16-s blocks (except for the final bin which was 20 s in duration). Bins were averaged across the three intertrial intervals. Figure 3D shows the mean (±SEM) percentage of time spent freezing during each bin. Repeated measures ANOVA revealed a main effect of condition [*F*(2,23) = 6.48, *p* < 0.05], a significant difference across bins [*F*(12,276) = 6.35, *p* < 0.001], but no bin–condition interaction [*F*(24,276) = 0.62, *p* > 0.05]. Post-hoc comparisons using Fisher’s PLSD revealed that Trace-LEC rats froze significantly less compared to Trace-Sham rats [*p* < 0.05] as well as Trace-MEC [*p* < 0.05]. However, Trace-MEC rats were not significantly different compared to controls [*p* > 0.05].

### 3.4. Delay Fear Conditioned Animals—Context Test

**Figure 4:**
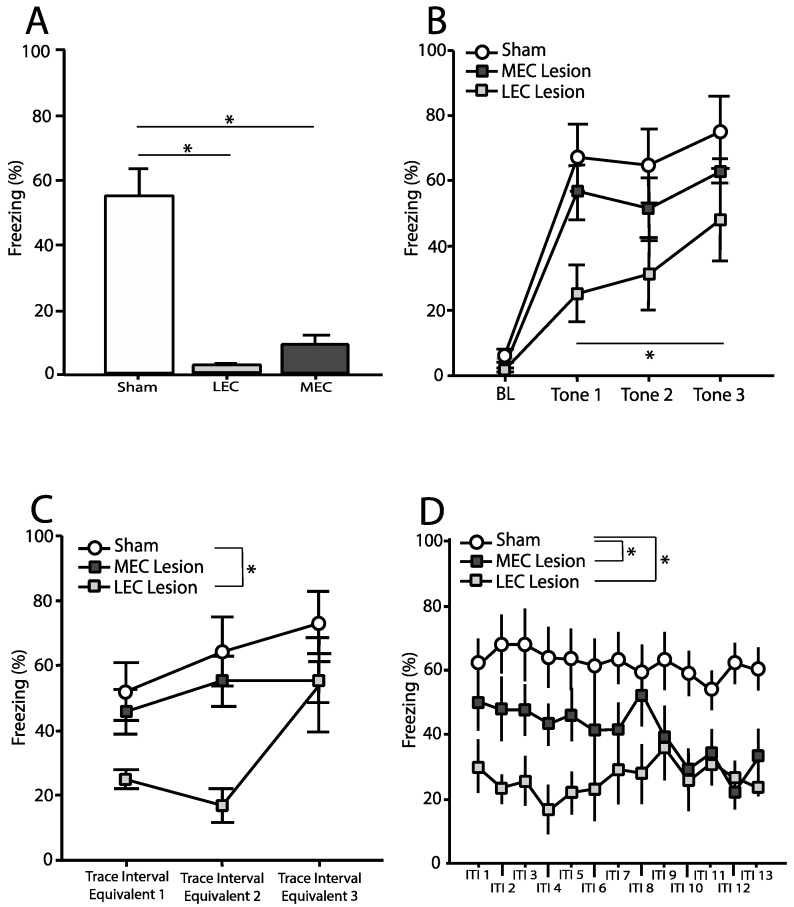
Mean (±SEM) percentage time spent freezing during the context test and tone test for rats trained using ***delay conditioning***. (**A**) Freezing during the entire 8-min context test. Both lesion groups show a deficit in freezing to context. (**B**) Baseline (BL) freezing taking place during the first 180 s of the tone test, prior to presentation of the first tone as well as freezing during each 16-s tone presentation. * Indicates that LEC lesioned rats froze significantly less during the tone compared to control rats. (**C**) Freezing during the 28-s trace interval equivalent following each tone presentation. There was a significant difference across trace interval equivalents and main effect of condition. LEC lesioned rats froze significantly less compared to control rats. (**D**) Freezing during each 212 s period following each trace interval divided into 16-s bins (with bin 13 being 20 s). There was a significant bin X condition interaction as well as a main effect of condition. Both LEC and MEC lesioned rats froze significantly less compared to control rats.

Freezing was measured during the entire 8-min context test. Figure 4A shows the mean (±SEM) percentage of time spent freezing during the context test for animals in each of the three conditions: Delay-Sham, Delay-MEC, Delay-LEC. A one-way ANOVA revealed a significant effect of condition on freezing to the context [*F*(2,19) = 27.93, *p* < 0.001]. Post-hoc comparisons using Fisher’s PLSD revealed that both Delay-LEC [*p* < 0.001] and Delay-MEC [*p* < 0.01] froze significantly less than Delay-Sham. However, Delay-LEC and Delay-MEC were not significantly different from one another [*p* > 0.05].

### 3.5. Delay Fear Conditioned Animals—Tone Test

#### 3.5.1. Baseline

Freezing was measured for the duration of the 180 s baseline period prior to the first tone and average percent freezing calculated. Figure 4B shows the mean (±SEM) percentage of time spent freezing during baseline. A one-way ANOVA revealed no significant effect of condition on freezing during the baseline period [*F*(2,19) = 2.78, *p* > 0.05].

#### 3.5.2. Tone

Freezing was measured for the duration of each 16 s tone. Figure 4B shows the mean (±SEM) percentage of time spent freezing during each tone. Repeated measures ANOVA revealed no significant differences across tones [*F*(2,38) = 3.39, *p >* 0.05] and no tone–condition interaction [*F*(4,38) = 0.3, *p* > 0.05]. However, there was a main effect of condition on freezing to the tone [*F*(2,19) = 4.02, *p* < 0.05]. Post-hoc comparisons using Fisher’s PLSD revealed that Delay-LEC rats froze significantly less than Delay-Sham [*p* < 0.01], but no difference between Delay-MEC and Delay-Sham [*p* > 0.05].

#### 3.5.3. Trace Interval Equivalent

Freezing was measured for the duration of each 28 s trace interval equivalent. Figure 4C shows the mean (±SEM) percentage of time spent freezing during each trace interval. Repeated measures ANOVA revealed a significant difference across trace interval equivalents [*F*(2,38) = 9.34, *p* = 0.001], but no trace interval–condition interaction [*F*(4,38) = 2.17, *p* > 0.05]. Additionally, there was a significant main effect of condition on freezing during the trace interval [*F*(2,19) = 4.47, *p* < 0.05]. Post-hoc comparisons using Fisher’s PLSD revealed that Delay-LEC rats froze significantly less compared to Delay-Sham rats [*p* < 0.01], but Delay-MEC rats did not differ from controls [*p* > 0.05].

#### 3.5.4. Intertrial Interval

Freezing was measured for the duration of each 212 s period following each trace interval. The intertrial intervals were binned into 16-s blocks (except for the final bin which was 20 s in duration). Bins were averaged across the three intertrial intervals. Figure 4D shows the mean (±SEM) percentage of time spent freezing during each bin. Repeated measures ANOVA showed no significant difference across bins [*F*(12, 228) = 1.64, *p* > 0.05], and no bin–condition interaction [*F*(24, 228) = 1.44, *p* > 0.05]. There was, however, a main effect of condition on freezing during the ITI [*F*(2,17) = 6.96, *p* < 0.01]. Post-hoc comparisons using Fisher’s PLSD revealed that both Delay-LEC rats [*p* < 0.01] as well as Delay-MEC rats [*p* < 0.05] froze significantly less compared to Delay-Sham rats. Additionally, Delay-MEC and Delay-LEC rats were not significantly different from one another [*p* > 0.05].

### 3.6. Trace Fear Conditioned Animals—Open Field

**Figure 5:**
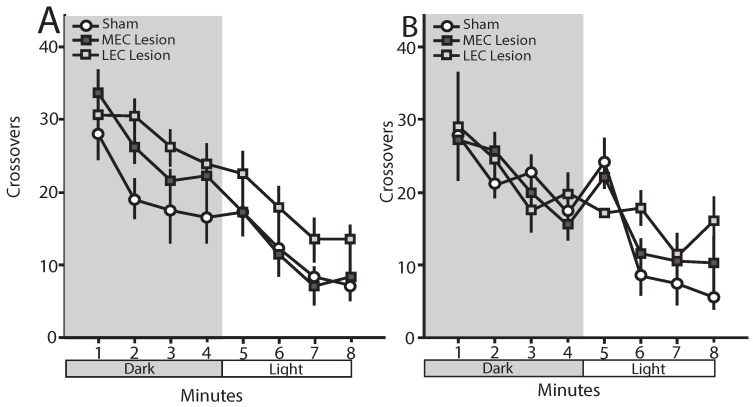
Mean (±SEM) number of crossovers in an open field during a 8-min test with four minutes of dark and four minutes of light. (**A**) Mean crossovers for rats given ***trace conditioning***. There was a significant difference in crossovers across minutes, but no main effect of condition. (**B**) Mean crossovers for rats given ***delay conditioning***. There was a significant difference in crossovers across minutes as well as a crossover X condition interaction. However, there was no main effect of condition.

The number of crossovers made during each minute of an 8-min open field test were measured. Figure 5A shows the mean (±SEM) number of crossovers by minute. Differences in number of crossovers made during dark versus light periods of the open field were analyzed separately using repeated measures ANOVAs. Across the 4 min of the dark, there was a significant effect of minute [*F*(3,69) = 7.41, *p* < 0.001], but no main effect of condition [*F*(2,23) = 3.146, *p* = 0.062] or minute–condition interaction [*F*(6,69) = 0.55, *p* = 0.715]. Across the 4 min of the light, there was a significant effect of minute [*F*(3,69) = 10.78, *p* < 0.001], but no main effect of condition [*F*(2,23) = 2.67, *p* = 0.09] or minute–condition interaction [*F*(6,69) = 0.058, *p* = 0.997]. Further, ANOVA revealed that the average number of crossovers during the dark was significantly higher than the average number of crossovers during the light [*F*(1,23) = 97.99, *p* < 0.001], but there was no main effect of condition [*F*(2,23) = 3.15, *p* = 0.062] nor a condition–lighting interaction [*F*(2,23) = 2.26, *p* = 0.128].

### 3.7. Delay Fear Conditioned Animals—Open Field

The number of crossovers made during each minute of an 8-min open field test were measured. Figure 5B shows the mean (±SEM) number of crossovers by minute. Differences in number of crossovers made during dark versus light periods of the open field were analyzed separately using repeated measures ANOVAs. Across the 4 min of the dark, there was a significant effect of minute [*F*(3,57) = 8.98, *p* < 0.001], but no main effect of condition [*F*(2,19) = 0.046, *p* = 0.955] or minute–condition interaction [*F*(6,57) = 0.914, *p* = 0.491]. Across the 4 min of the light, there was a significant effect of minute [*F*(3,57) = 15.70, *p* < 0.001] and a minute–condition interaction [*F*(6,57) = 3.07, *p* < 0.05], but no main effect of condition [*F*(2,19) = 1.38, *p* = 0.275]. Further, ANOVA revealed that the average number of crossovers during the dark was significantly higher than the average number of crossovers during the light [*F*(1,19) = 107.99, *p* < 0.001], but there was no main effect of condition [*F*(2,19) = 0.555, *p* = 0.58] nor a condition–lighting interaction [*F*(2,19) = 1.52, *p* = 0.245].

## 4. Discussion

The present results suggest that both the LEC and MEC are necessary for the expression of contextual fear, as lesioning of either structure led to a robust deficit in context freezing following both trace and delay fear conditioning. This is consistent with studies showing that lesions of either the entire EC [7] or the MEC produces deficits in freezing to context [12,31], and extends those findings to show that selective lesions of LEC produce similar deficits in contextual fear memory expression.

In addition, our data indicate that auditory fear memories depend upon the LEC selectively. Specifically, freezing during the tone (in delay conditioned rats) and the trace interval (or equivalent) was attenuated following lesions of the LEC, but not MEC. In contrast, pre-training lesions of the entire EC have produced no deficits in freezing following delay conditioning but did produce deficits following trace conditioning [10]. Much of the data looking at post-training manipulations come from studies of eyeblink conditioning. It has been shown that reversible inactivation of the LEC following trace eyeblink conditioning produces deficits [18,19], but not when the MEC is inactivated [18]. While data from eyeblink conditioning seem to produce a more robust functional heterogeneity, it is possible that trace and delay eyeblink conditioning involves different brain regions and/or mechanisms compared to trace and delay fear conditioning making direct comparisons difficult. Finally, freezing that was maintained throughout the ITI in both trace and delay conditioned animals was robustly attenuated following lesions of the LEC, whereas lesions of the MEC produced either no effect (trace conditioning) or a modest effect (delay conditioning) on freezing during the ITI. Taken together, these data support the hypothesis that MEC primarily mediates contextual components of fear memory expression. However, the data do not support the hypothesis that the role of the LEC in fear memory is in retrieval and/or expression of the temporal association between stimuli separated in time. If LEC played a critical role in the expression of temporal association, one would predict significant freezing deficits in trace, but not delay, conditioned animals. However, the current data show significant LEC lesion-induced tone-related deficits following both trace and delay conditioning.

While the data from the current experiment do not show the clear functional heterogeneity of the LEC and MEC in trace vs. delay conditioning, the differences in freezing over the entire tone test may provide novel insights into how these EC subregions regions contribute to the expression of auditory cued fear and are potentially more revealing of the underlying mechanisms than measuring total freezing across the entire duration, or only during select periods. If the same mechanisms were controlling freezing for the entirety of the test session, then one would expect to find the same patterns of impairment during each time window. However, previous data from the hippocampus literature has shown that this is not the case. Following post-training lesions of the dorsal hippocampus with delay conditioning, both sham and lesioned rats showed similar levels of freezing during the presentation of a 16-s tone during testing. If this measure were the only observation, it would have been concluded that there were no differences in these groups. However, when comparing levels of freezing once the tone has shut off, a difference between the groups is revealed [21,22]. These differences demonstrate that it is important to investigate not just whether, but when, animals are freezing. Furthermore, this also reveals that freezing during the tone and freezing after the tone may be mediated by two distinct mechanisms. This same pattern is seen in rats administered post-training infusions of the AMPA/kainite glutamate receptor antagonist, CNQX, restricted to either the CA1 region or the dentate gyrus of the dorsal hippocampus. Those rats with CNQX targeted to the CA1 looked identical to controls for freezing during the tone and trace interval, but significant differences were revealed when measuring freezing during the ITI [31]. One hypothesis is that freezing during the tone test is mediated by both contextual memory and memory for the discrete tone that is distinct from context. While both context and tone become associated with footshock during fear conditioning, context and tone also can become associated with one another and this memory may be driving the freezing observed during the intertrial interval [21,22,32]. Likewise, electrophysiological recordings from the LEC during a trace eyeblink conditioning task in rodents have shown that firing in the LEC is selective for not only the modality of the conditioned stimulus, but the spatial location in which it was presented [33]. Thus, it may be that the tone presentation during testing retrieves both the tone–footshock association as well as the tone–context association, and when the tone is terminated, post-tone freezing is mediated more by memory for the conditioning context. A similar pattern is seen during the ITI in trace and delay conditioned rats with hippocampus lesions [21,22]. These data combined with the significant deficits in freezing to the context suggest that LEC may, in fact, mediate some contextual component of the fear memory and that this is needed to maintain freezing during the post-tone period. Furthermore, the lack of an effect on freezing during the presence of the tone suggests that the LEC is undergoing processes independent from the hippocampus and not simply serving as an information relay to and from the hippocampus.

The above interpretation is consistent with other studies showing LEC involvement in memory for conjunctive representations. Rats with lesions restricted to the LEC were shown to be impaired on an item novelty detection task with four objects, but not impaired on a contextual novelty detection task [26]. Similarly, LEC-lesioned rats were impaired on an object-context recognition task in which rats were shown a matching pair of novel objects in a novel context in one session, a new pair of novel matching objects in a novel context in a second session, and then were tested by being placed back in one of the contexts with objects from both contexts present. In contrast, these same rats were unimpaired on object recognition and context recognition [34]. It has been demonstrated that lesions of the LEC did not impair the ability to perform a spatial water maze task, but did impair both spatial (moving one of four objects following habituation) and nonspatial (replacing one of four objects with an entirely new object) versions of an object exploration task. In contrast, LEC lesions had no effect when only two objects were used [35]. When rats were trained to dig for a food reward in local spatial framework with only intramaze cues vs. a global task using extramaze cues, lesions to the LEC resulted in slower learning in the local task compared to the global, but MEC lesions resulted in rats learning the local task faster than global [36]. It has also been suggested that the deep layers of the LEC are recruited under higher memory load conditions based on higher levels of Arc activity in rats trained on a non-spatial, 10-odor delayed non-match to sample task compared to controls, but not a 5-odor version [37].

One potential caveat to these conclusions is that some of the LEC lesions included damage to the perirhinal cortex. Perirhinal cortex damage has been shown to lead to impairments in both acquisition and expression of contextual fear [38,39,40]. Additionally, it has been demonstrated that post-conditioning lesioning of the perirhinal cortex leads to an impairment in fear-potentiated startle in response to an auditory cue in a novel context [41,42]. However, when looking at effects of perirhinal manipulations on trace and delay fear conditioning, it has been shown that infusion of the muscarinic receptor antagonist, scopolamine, into the perirhinal cortex lead to a significant reduction in freezing in trace, but not delay, conditioning [43]. Although there were methodological differences between that study and the current one that make it difficult to assess potential parallels to our post-tone freezing, particularly regarding the duration of the test tone, the lack of scopolamine effect on delay fear suggests that our LEC lesion effects follow a distinct pattern from those observed following manipulation of the perirhinal cortex.

Lesions of the hippocampus have been suggested to produce locomotor hyperactivity [44]. To assess whether our lesion deficits in freezing might be attributable to locomotor hyperactivity, we assessed activity in an open field under two lighting conditions [25]. We did not observe differences between either lesion condition and sham controls during either the dark or light gradient conditions. Along with our data showing that both LEC and MEC lesioned rats freeze at comparable levels to controls during distinct components of the fear testing, this suggests that the lesion deficits that we see are not attributable to hyperactivity.

## 5. Conclusions

Data from the present study support the hypothesis that the MEC’s role in trace and delay fear conditioning is primarily restricted to contextual aspects of the memory as manipulations of the MEC only produced effects on freezing to the context and freezing during the post-tone period, which is hypothesized to have a contextual component. Our data do not support a role for LEC in forming/maintaining a temporal association between two non-contiguous stimuli, as lesions to the LEC produced a deficit in tone freezing only in delay conditioned animals. Given the consistent effect of LEC lesions on context fear, freezing during trace interval and equivalent, and ITI, it appears that LEC may also use contextual information in its contributions to fear memory expression, possibly through mediating post-tone freezing via conjunctive representation.

## Figures and Tables

**Table 1:** Stereotaxic coordinates for LEC and MEC lesions taken from bregma (in mm).

**LEC**	**Site 1**	**Site 2**	**Site 3**	**Site 4**
AP	−4.8	−4.8	−6.8	−6.8
ML	±6.8	±6.8	±6.5	±6.5
DV	−7.8	−8.8	−7.5	−8.5
**MEC**	**Site 1**	**Site 2**	**Site 3**	
AP	−8.3	−8.8	−8.8	
ML	±4.8	±6.2	±6.2	
DV	−7	−7	−8	

## Data Availability

Upon publication of the manuscript, the data presented in this paper will be openly available in Scholarly Commons at [doi: TBD], reference number [reference number: TBD].
